# How (Not) to Measure Loneliness: A Review of the Eight Most Commonly Used Scales

**DOI:** 10.3390/ijerph191710816

**Published:** 2022-08-30

**Authors:** Marlies Maes, Pamela Qualter, Gerine M. A. Lodder, Marcus Mund

**Affiliations:** 1 Interdisciplinary Social Science: Youth Studies, Utrecht University, 3584 CS Utrecht, The Netherlands; 2 Manchester Institute of Education, The University of Manchester, Manchester M13 9PL, UK; 3 Department of Developmental Psychology, Tilburg University, 5037 AB Tilburg, The Netherlands; 4 Personality Psychology and Psychological Assessment, Klagenfurt University, 9020 Klagenfurt, Austria

**Keywords:** loneliness, measurement, psychometrics, factor structure, reliability, measurement invariance, loneliness types

## Abstract

Loneliness affects well-being and has long-term negative impacts on physical and mental health, educational outcomes, and employability. Because of those current and long-term impacts, loneliness is a significant issue for which we need reliable and appropriate measurement scales. In the current paper, psychometric properties of the eight most commonly used loneliness scales are reviewed both descriptively and meta-analytically. Results suggest that for many of the scales, the psychometric properties are promising. However, for some psychometric features, especially test-retest reliability and measurement invariance, evidence is rather scarce. Most striking, however, is the fact that all of the scales included items that do not measure loneliness. Surprisingly, for many (sub)scales, this was even the case for about half of the items. Because our measures are the foundation of our research work, it is crucial to improve the way loneliness is being measured.

## 1. Introduction

*Let me tell you this: if you meet a loner, no matter what they tell you, it’s not because they enjoy solitude. It’s because they have tried to blend into the world before, and people continue to disappoint them*.

(Picoult 2009, p. 156) [1]

Most people, no matter how old they are or where they live, have probably experienced loneliness at some point in their lives [2]; most of us have had to manage loneliness experiences and cope with the associated negative affect. In 1981, Perlman and Peplau defined loneliness as “the unpleasant experience that occurs when a person’s network of social relations is deficient in some important way, either quantitatively or qualitatively” [3] (p. 31). For many individuals, loneliness is a temporary experience. However, for other people, difficulties arise when attempting to fulfill their social needs, and feelings of loneliness endure [4]. Enduring feelings of loneliness are of high concern because they are associated with both current well-being and future mental and physical health problems (for example, see [5,6,7,8], this special issue). For example, people who experience loneliness have been found to experience more anxiety and depressive symptoms, have more sleep problems and cardiovascular incidents, become ill more quickly, and die at an earlier age (for reviews, see [9,10,11]). Loneliness is also a force for downward mobility [12], with lonelier young adults obtaining lower academic grades by age 18 years compared to their peers and being more likely to be out of work and education. Those experiencing prolonged loneliness from childhood through late adolescence appear to be particularly at risk [13]. Because loneliness is seen to have such important and damaging impacts, a number of measures have been developed to assess loneliness. 

Measures are the foundation of our research work, and hence, form the basis of our conclusions and recommendations. We use measures to obtain our findings; therefore, it is crucial to understand their quality. To assess loneliness, both single- and multi-item measures have been used. Most of the single-item measures are direct measures, that is, they include the specific word “lonely” or “loneliness” [14]. Single-item measures come in great variety and have been criticized for several reasons (see, for a further discussion on this issue, [14]). In the present review paper, we focus on the eight most commonly used multi-item measures of loneliness and discuss their strengths and weaknesses and their most important conceptual and psychometric features. Specifically, we focus on the University of California Los Angeles Loneliness Scale (UCLA; [15]), the Children’s Loneliness Scale (CLS; [16]), the Rasch-Type Loneliness Scale (RTLS; [17]), the Social and Emotional Loneliness Scale for Adults (SELSA; [18]), the Differential Loneliness Scale (DLS; [19]), the Loneliness and Aloneness Scale for Children and Adolescents (LACA; [20]), the Relational Provisions Loneliness Questionnaire (RPLQ; [21]), and the Peer Network and Dyadic Loneliness Scale (PNDLS; [22]). None of these questionnaires, except for one item of the CLS, contain the words “lonely” or “loneliness”; therefore, they can be considered as indirect measures of loneliness.

We focus on three main aspects of those scales. First, we examine what type of loneliness each of these scales assesses. More specifically, we examine to what extent the items in each scale measure the differential types of loneliness (i.e., emotional and social loneliness). Second, we examine the psychometric properties of the scales, including factor structure, reliability, and measurement invariance. Third, we discuss and meta-analytically investigate how the different measures relate to each other, which will help when interpreting research findings obtained in different studies using different measures. Taken together, this comprehensive comparison will help researchers make suitable choices for a loneliness measure in their future research.

## 2. Materials and Methods

For the different research aims of the present review paper, different methods are employed. Most data were obtained from the MASLO (“Meta-Analytic Study of Loneliness”) project. The MASLO project is a large database containing studies that have used one of the eight main loneliness questionnaires (i.e., the UCLA, CLS, RTLS, SELSA, DLS, LACA, RPLQ, and PNDLS). The initial search for creating the MASLO database was conducted in four literature databases, PsychInfo, ERIC, PubMed, and Web of Science, using key terms that reflected the names of the loneliness measures. For example, for the UCLA, we used the search strings (“UCLA Loneliness Scale” or “UCLA Loneliness Questionnaire”) and ((UCLA) and (lonel* or “perceived social isola*”)). A full list of key terms can be found at the Open Science Framework (https://osf.io/tzg32/). Only empirical journal reports, books, and book chapters were included. Moreover, only studies written in English, Dutch, German, or French were included. Further details on the systematic literature search have been described elsewhere [23]. After the 2016 update of the literature search, 2632 reports were read in-depth, and 2318 reports were coded and included in the MASLO database (7 reports could not be retrieved, and 307 reports did include one of the eight loneliness questionnaires, but did not provide any further information on loneliness). 

For the 2318 reports that were coded and included in the MASLO database, a standardized, pre-piloted coding form and manual was used to extract the data. Undergraduate and graduate students in psychology were trained by the first author to code the reports until they reached a sufficient level of expertise. All reports coded by the students were checked by the first author to verify that the rules described in the manual had been applied correctly and no information was missed. The extracted information includes participant characteristics (e.g., the age, gender, clinical, ethnic, and socioeconomic status of the participants), the country the study was conducted in, and loneliness questionnaire characteristics (e.g., the questionnaire used, the number of items and response categories, the language the items are written in, correlations among subscales, Cronbach’s alpha, test-retest reliability and its time interval, and the mean loneliness score). 

In the present paper, we report on the coding of the items of the eight questionnaires (according to emotional and social loneliness), on descriptive reviews (on factor structure and measurement invariance), and on meta-analytic reviews (on reliability scores, test-retest reliability scores, and correlations among loneliness (sub)scales). The descriptive reviews of the factor structure and the meta-analytic estimates of internal consistency, test-retest reliability, and correlations among loneliness (sub)scales are based on the 2016 update of the MASLO database. For the descriptive review of the measurement invariance of the included scales, we conducted an additional literature search, as the issue of measurement invariance has gained attention in recent years, and we wanted to capture possible new evidence regarding this particular aspect. The pre-registration of the current study can be found here: https://www.crd.york.ac.uk/prospero/display_record.php?RecordID=136558.

### 2.1. Coding of Items According to Different Types of Loneliness

Researchers have increasingly advocated a multidimensional conceptualization of loneliness, where they distinguish among various hypothesized manifestations of loneliness [24]. The argument is that different social relationships may fulfill different social needs. For example, a relationship with a romantic partner may fulfill one’s need for intimacy, while relationships with friends may meet one’s needs for affiliation and social integration. Thus, it is likely that some of those social needs are fulfilled whereas others are not met. According to the multidimensional view on loneliness, those different types of unfulfilled needs would lead to different types of loneliness [24,25]. 

Several researchers have adhered to this multidimensional view of loneliness. However, different research teams have proposed dissimilar distinctions of loneliness types and developed different loneliness measures. These different loneliness measures are partly overlapping, but partly assess different loneliness types. Moreover, it is often not clear which loneliness types are captured by which measure, and different labels have been used for the same loneliness type, making it difficult to grasp how the different measures (and the research findings obtained with them) relate to each other. In this section, we propose an overarching conceptual framework, and, when reviewing the different measures, we show how each measure relates to this conceptual framework.

A first broad distinction that is often made in the loneliness literature is that of emotional and social loneliness. This distinction originates from the social needs perspective of Weiss, according to which different types of social relationships may fulfill different social needs [26]. Even though a distinction between emotional and social loneliness has often been made, consensus on a clear definition of those two types seems to be missing. As Weiss ([27], p. 12) himself stated: “*Having now suggested how little I feel I truly understand about the loneliness of emotional isolation, it seems appropriate to say that I feel even less confident about my understanding of the loneliness of social isolation*.” Based on a comparison, integration, and in-depth discussion of the different definitions of emotional and social loneliness that have been used in the literature, we propose the following two definitions. *Emotional loneliness* arises when a person perceives to lack the relationship provisions of close emotional attachments, including emotional support, affection, and intimacy. Emotional loneliness involves the feeling of not being close to people, of lacking people that really know and understand you, and who are there for you when you need it. *Social loneliness* arises when a person perceives they lack the relationship provisions of a network of contacts, including social integration and belongingness. Social loneliness involves the feeling of not being in tune with the people around you, of lacking people to talk to and spend time with, who you can ask for (instrumental) help, people you feel to belong to. 

In addition to those two broader types of loneliness, one could distinguish between other relationship-specific types of loneliness. People can experience emotional loneliness, for example, in their relationship with their best friend, but also in their relationship with their romantic partner or a parent. Similarly, people may experience social loneliness, for example, in their relationships with friends, but also in their relationships with family. 

To be able to clarify which types of loneliness are captured by which measures, we independently coded the items of the eight loneliness scales, indicating whether they reflected emotional or social loneliness. Next, three undergraduate students independently coded all items based on our definitions of emotional and social loneliness. During this process, we decided an additional category was needed to reflect items that may be related to loneliness, but do not seem to assess any formal definition of loneliness (e.g., “I am an outgoing person”). The three student coders reached good inter-rater agreement with a Fleiss Kappa of 0.627. We, the authors, also reached a consensus about which items do not measure loneliness; when removing those items from the lists of coded items, inter-rater agreement between the three independent student coders was even higher (Fleiss Kappa = 0.717). When reviewing the different loneliness measures, we describe for each measure, based on the coding, the percentage of items that assess emotional and social loneliness, respectively. Moreover, we discuss whether the items refer to specific relationships (e.g., with friends or family) or to people in general.

### 2.2. Descriptive Reviews

First, for each loneliness questionnaire, we noted how the original development papers for each scale defined loneliness. We then moved on to review existing evidence on factor structure and measurement invariance. Regarding the factor structure of the different loneliness measures, we reviewed evidence from both exploratory and confirmatory factor analyses. Regarding measurement invariance, we discuss both metric and scalar invariance. Metric invariance (i.e., factor loadings being equal across groups) is needed to meaningfully compare associations between variables across groups. Scalar invariance (i.e., both factor loadings and intercepts being equal across groups) is needed to meaningfully compare means across groups. Moreover, because measurement invariance is increasingly tested in the literature, we conducted an additional search in Google Scholar to retrieve loneliness studies published in 2016 or later that used one of the eight standardized loneliness measures and examined measurement invariance.

### 2.3. Meta-Analytic Reviews

To meta-analytically review reliability scores of the different loneliness measures, we conducted Reliability Generalization studies, using Cronbach’s alpha as an indicator [28,29]. Because Cronbach’s alpha by its nature follows a skewed distribution, we used Bonett-transformed alphas and their corresponding sampling variance [30]. We assessed evidence of publication bias with an extension of Egger’s regression test by adding the sampling variance as a moderator to the model. If there was systematic variance between studies’ reliability scores, we examined the moderating effect of several study and sample characteristics, that is, the age, gender, and clinical status of the participants, the country the study was conducted in, whether a translated version of the measure has been used, and whether the original version of the scale or a version with an adapted number of items or response categories was used. The specific categories that were coded for each moderator are described in the pre-registration documentation (see https://www.crd.york.ac.uk/prospero/display_record.php?RecordID=136558). However, oftentimes, fewer than five studies per category were available, which led to the merging of some of the categories. 

To meta-analytically review test-retest reliability scores, we selected studies that reported a correlation between two scale-scores (using the same instrument) that were a maximum of one month apart. Studies in which an intervention occurred in between measurement occasions were not included. We transformed all correlations to Fisher’s *Z*r. When fewer than five studies were available, we used a fixed-effect model, and when five studies or more were available, we used a random-effects model. To facilitate interpretation, we converted the results back to Pearson correlations *r*.

To meta-analytically review correlations, both between subscales within a particular loneliness questionnaire and between (sub)scales of different loneliness questionnaires, we transformed all correlations to Fisher’s *Z*r to conduct the analyses and backtransformed them to Pearson correlations *r* for reporting the results. 

Regarding all meta-analyses described above, when studies reported on multiple effect sizes, we conducted a three-level meta-analysis, including sampling variance, within-study variance, and between-study variance, to account for the possible dependency of the data. Analyses were conducted with the metafor package in R [31]. Analysis scripts and data used in the analyses are accessible at https://osf.io/5wn93/.

## 3. Results

### 3.1. The University of California Los Angeles Loneliness Scale (UCLA)

The UCLA consists of 20 items [15,32,33]. There are four response categories, ranging from 1 (never) to 4 (often), hence, the UCLA assesses the frequency of loneliness experiences. Common brief versions include (but are not limited to) the 8-item version of Roberts et al. ([34]; R-ULS), the 8-item version of Hays and DiMatteo ([35]; ULS-8) (both 8-item versions do not include the same 8 items), the 4-item version of Russell et al. [15], and the 3-item version of Hughes et al. [36]. There are other adaptations of the items, but it is not always clear which items were used in a particular study, and sometimes items from the different adaptations have been combined [37]. When discussing loneliness, the original development paper(s) for the UCLA emphasizes the quantity of the relationships, and places loneliness at the opposite end of the continuum of feeling crowded, as the experience of having too few social relationships (e.g., [33]).

The scale was developed in English for use with (young) adults. Of all studies included in the MASLO database, 64.24% used this scale, making it the most used loneliness questionnaire. The scale is mostly used with college students (35.11%) and adults (33.70%), to a lesser extent with adolescents (17.28%) and older people (13.30%), and rarely with children (0.61%). The UCLA has been translated in many languages and is used across the world, but mainly in North America (59.69%), Europe (20.06%), and Asia (17.04%). 

The UCLA was developed as a unidimensional scale and is mostly used as such in the literature. Some researchers, however, have distinguished between different loneliness types, although there is no consensus in the literature about which types to differentiate. Moreover, even when the same number of factors are used in different articles, often those factors do not include the same set of items. Based on our coding of items (see Table 1), seven items (35% of the total number of items) measure emotional loneliness and eleven items (55%) measure social loneliness. We do not regard the remaining two items as measuring loneliness. One item refers to “friends”, but the other items are not relation-specific and mostly refer to “people”.

#### 3.1.1. Evidence on the Factor Structure of the UCLA

The factor structure of the UCLA has been frequently examined, but consensus on the best fitting structure is still lacking. Based on exploratory factor analyses, several factor solutions have been proposed. A 1-factor solution has been proposed in a sample of Greek female university students [38], a sample of German adults [39], samples of Canadian university students and older adults [40], and a sample of South African students [41], though in several cases, one or more items were omitted. In most studies, however, a factor solution containing multiple factors has been proposed, most often a 2- or 3-factor solution. In three samples, a 2-factor solution was found, reflecting the direction of item wording (i.e., negatively versus positively worded items), including New Zealand adults [42], Danish adolescents [43], and Chinese adolescents [44]. In other studies, a 2-factor solution was also proposed, but in those cases the factors did not (completely) reflect item wordings. Usually, the two found factors were labelled to reflect emotional and social loneliness, but at the same time, those factor solutions did not include the same set of items [45,46,47,48,49]. A 3-factor solution also has been frequently found, including a sample of US adolescents [46], samples of US university students [50,51,52,53], and a sample of German adults [54]. Those factors have been labeled with different, but sometimes overlapping labels, and none of the found factor solutions represent the same set of items. Less frequently, 4- and 5-factor solutions have been proposed [35,55,56].

Based on confirmatory factor analyses, excellent fit was found for a 1-factor model in a sample of US university students [57]. However, in most other studies, the 1-factor model resulted in insufficient or poor model fit, and multiple factor models have been proposed instead [56] (e.g., [58,59]). Better model fit was found in several studies when item wording was taken into account [32,58,59,60], but not in all samples [56]. In two other samples, a 3-factor solution was proposed, where all negatively worded items loaded on one factor, and the positively worded items loaded on the two other factors [52,61].

**Table 1:** Coding of Items of the University of California Los Angeles Loneliness Scale (UCLA).

Item	Emotional Loneliness	Social Loneliness	No Loneliness
I feel in tune with the people around me		X	
I lack companionship		X	
There is no one I can turn to	X		
I do not feel alone		X	
I feel part of a group of friends		X	
I have a lot in common with the people around me		X	
I am no longer close to anyone	X		
My interests and ideas are not shared by those around me		X	
I am an outgoing person			X
There are people I feel close to	X		
I feel left out		X	
My social relationships are superficial	X		
No one really knows me well	X		
I feel isolated from others		X	
I can find companionship when I want it		X	
There are people who really understand me	X		
I am unhappy being so withdrawn			X
People are around me but not with me		X	
There are people I can talk to		X	
There are people I can turn to	X		

#### 3.1.2. Evidence on Measurement Invariance of the UCLA

Several studies have examined measurement invariance, mostly focusing on gender. Metric invariance has been established in a sample of French-Canadian teachers [61] and in a sample of US adults [62]. In a sample of Chinese adolescents, partial scalar invariance across gender was established [63], and in two samples of Belgian adolescents, scalar invariance was established [59,64]. In a sample of Iranian University students, based on Differential Item Functioning, invariance across gender was also found [65], and in two samples of UK adults and UK older adults, strict invariance across gender was established [66]. In addition, strict invariance has been established in US adolescents, comparing participants endorsing a nonheterosexual orientation and any non-cisgender identity, participants endorsing a nonheterosexual orientation and a cisgender identity, and participants endorsing a heterosexual orientation and a cisgender identity [67]. 

Measurement invariance across age has also been examined, with results indicating scalar invariance, for different brief versions of the UCLA, in a sample of German adults [68], US adults [62], and UK adults [37]. The latter study also examined measurement invariance for the full 20-item scale, but only partial invariance could be established. Regarding comparisons across cultures, scalar invariance has been established between US and German older people [69] and between adults from Germany, Indonesia, and the US, but only for the 6-item version of the scale [70]. In the same study, for the 8-item version, invariance could not be established, and for the 20-item version, it was not tested, as no satisfactory model fit could be reached. In a sample of US adults, metric invariance was further established across race, marital status, employment status, income, and education [62], and scalar invariance was established between US adults with schizophrenia or schizoaffective disorder and non-psychiatric comparison subjects [71]. In a sample of French-Canadian teachers, metric invariance was established between elementary and high school teachers [61]. Longitudinal invariance, with measurements two weeks apart, was examined in a sample of Canadian adults and older people, but no metric invariance could be established [56].

#### 3.1.3. Evidence on the Reliability of UCLA Scores

For the UCLA, a Reliability Generalization study is already available [72]. The results of this study by Vassar and Crosby revealed a mean internal consistency reliability coefficient of 0.87 (*SD* = 0.06, *k* = 80). Several moderators were tested as well. Results revealed a positive relation between variability (i.e., score standard deviation) and reliability, and revealed that substantive articles tended to yield higher reliability estimates on average than articles focusing on measurement development or psychometric evidence. Furthermore, the univariate, but not multivariate, analysis of Vassar and Crosby showed that studies that included adolescent samples yielded lower reliability estimates on average than studies that included samples of older populations.

#### 3.1.4. Evidence on Test-Retest Reliability of UCLA Scores

Five studies reported the correlation between UCLA scores that were assessed maximum one month apart. Those correlations ranged from *r* = 0.46 to *r* = 0.91, with the intervals between assessments ranging from 1 to 4 weeks (Median = 2 weeks). Back-transformed results from the random-effects meta-analysis yielded an estimated mean effect size of *r* = 0.83, 95% CI [0.79, 0.86].

### 3.2. The Children’s Loneliness Scale (CLS)

Different names have been used to refer to this questionnaire, mostly “Children’s Loneliness Scale” and “Loneliness and Social Dissatisfaction Questionnaire”, but the questionnaire is also frequently referred to without a specific name (e.g., “loneliness scale”, “loneliness questionnaire”, and “asher loneliness scale”). The CLS consists of 24 items, of which 16 items are used to assess loneliness, whereas the other 8 items reflect “filler items” and are not used (e.g., “I like music”). The items can be answered on a 5-point scale ranging from 1 (always true) to 5 (not true at all), reflecting both frequency and agreement. Brief versions are also used in the literature, sometimes referred to as measures of ‘pure’ loneliness, but different versions have appeared (e.g., containing 3, 4, or 5 items) and not much validation work has been performed so far on those versions. The original development papers for the CLS give no clear definition of loneliness but describe the loneliness questionnaire as a subjective alternative for sociometric measures of social status and popularity (e.g., [16]).

The CLS was developed in English, for use with children. Of all the studies included in the MASLO database, 20.89% used this scale. The scale is mostly used with children (65.73%), but also with adolescents (34.09%). One study used the scale in a sample of adults diagnosed with intellectual disabilities [73]. The CLS has been translated in many languages and is used across the world, mainly in North America (62.90%) and Asia (22.41%), and to a lesser extent in Europe (9.60%), Australia (4.90%), and South America (0.19%). 

The CLS has been developed as a unidimensional scale and is mostly used as such in the literature. Subscales reflecting different types of loneliness, however, have also been proposed, but no consensus has been reached in this regard (see below, where we discuss evidence on the factor structure of the CLS). Based on our coding of items (see Table 2), two items (13% of the total number of items) measure emotional loneliness, five items (31%) measure social loneliness, and one item was not coded, as it could reflect both emotional and social loneliness (i.e., “I’m lonely”). We do not regard the remaining eight items (50%) as measuring loneliness (e.g., “I’m good at working with other children”). Some items do not refer to specific relationships, but most items (*n* = 10) are about other children or friends [16]. In a later version of the scale, the items were adapted by supplying a clear school focus to each of them (e.g., “I have nobody to talk to” was changed into “I have nobody to talk to in class”) [74].

#### 3.2.1. Evidence on the Factor Structure of the CLS

Based on exploratory factor analyses, a 1-factor solution was found in three samples of US children [16,74,75] and in a sample of Turkish children [76]. In a sample of US adolescents, a 2-factor solution was found, reflecting positively and negatively worded items [77]. 

Based on confirmatory factor analyses, the 1-factor model showed poor model fit in a sample of Turkish children [76], but when one item was excluded, model fit was acceptable. However, other studies, also revealing poor model fit for the 1-factor model, proposed other solutions. Based on a sample of African and Hispanic American children, a 2-factor solution was proposed that represented item wording [78]. However, in two other studies, including US children and adolescents [79] and Belgian children [80], a better fit was found for a 1-factor solution with correlated error terms to account for item wording than for the 2-factor model. In contrast, this 1-factor model with correlated error terms only showed sufficient model fit in a sample of Belgian adolescents when adding an error correlation between two items that were very similar in wording [59]. In a sample of US children, good model fit was found for a model with two first order factors (based on item wording), one second order factor (reflecting all items), and four error correlations [81].

#### 3.2.2. Evidence on Measurement Invariance of the CLS

Measurement invariance has been tested across gender, age, culture, and time. Regarding gender, scalar invariance has been established in a sample of US children [79] and in a sample of Belgian adolescents [59]. In a sample of US adolescents, only partial metric and scalar invariance across gender could be established [79]. Regarding age, scalar invariance was established in a sample of US children and adolescent boys, but only partial metric and scalar invariance was established in a sample of US children and adolescent girls [79]. Measurement invariance across cultures has, to our knowledge, only been examined in a sample of US children and adolescents, in which scalar invariance was found for the four examined group comparisons, i.e., African American children vs. European American children, Asian American children vs. Latin American children, African American adolescents vs. European American adolescents, and Asian American adolescents vs. Latin American adolescents [82]. Longitudinal invariance has also been examined in US samples only. Full metric and partial scalar invariance across Grade 3 and Grade 5 was established for a sample of US children [81], and metric invariance (scalar invariance was not examined) across three assessment waves with 18-month intervals was established in a sample of US adolescents using a 7-item version of the CLS [83].

#### 3.2.3. Evidence on the Reliability of CLS Scores

Back-transformed results from the three-level meta-analysis, based on 319 effect sizes, revealed a mean internal consistency reliability coefficient of 0.86, 95% CI [0.85, 0.87]. Results regarding the moderator analyses can be found in Appendix A. Most moderators did not significantly affect the reliability estimates, including the moderators General population, Gender, Country, and Translated. The moderators Age and Adaptation were significant. Results revealed that reliability estimates were slightly lower on average for samples of children (mean estimate = 0.86, 95% CI [0.85, 0.86]) than for samples of adolescents (mean estimate = 0.87, 95% CI [0.86, 0.88]). In addition, reliability estimates were somewhat higher on average for studies in which the original scale was used (mean estimate = 0.88, 95% CI [0.87, 0.88]) than for studies in which adapted versions were used, including brief versions (mean estimate = 0.84, 95% CI [0.82, 0.85]), versions including all items but with another number of response categories (mean estimate = 0.81, 95% CI [0.78, 0.84]), and versions with another number of items and another number of response categories (mean estimate = 0.81, 95% CI [0.77, 0.83]). The publication bias test did not reveal evidence for bias (*p* = 0.538).

#### 3.2.4. Evidence on Test-Retest Reliability of CLS Scores

Seven studies reported the correlation between CLS scores that were assessed a maximum one month apart. Those correlations ranged from *r* = 0.75 to *r* = 0.93, with the intervals between assessments ranging from 1 to 4 weeks (Median = 2 weeks). Back-transformed results from the random-effects meta-analysis yielded an estimated mean effect size of *r* = 0.76, 95% CI [0.74, 0.77].

### 3.3. The Rasch-Type Loneliness Scale (RTLS)

The RTLS, also frequently referred to as the De Jong Gierveld Loneliness Scale (DJGLS), consists of 11 items with answering categories reflecting agreement. According to the developers of this scale, response options include five categories: “yes!”, “yes”, “more or less”, “no”, and “no!” (or, alternatively, strongly (dis)agree, (dis)agree, and more or less) or three categories: “yes”, “more or less”, and “no”. Next, the scores are dichotomized so that the scale scores will range from 0 to 11. For positively worded items, the answers “no!”, “no”, and “more or less” are coded as 1, as they are considered an expression of loneliness. For negatively worded items, “yes!”, “yes”, and “more or less” are coded as 1. In other words, the answer “yes” is coded as 1 (lonely) only for the negatively worded items, the answer “no” is coded as 1 (lonely) only for the positively worded items, and the answer “more or less” is coded as 1 (lonely) for all items (both positively and negatively worded ones). This approach of dichotomizing the items has been questioned (e.g., [84]), and it is unclear whether this dichotomization was conducted the same way in all studies using the RTLS. 

Compared to the original scale, the items presented in the manual of the RTLS [85] are worded in a slightly different way. Those adapted items are the same as presented in De Jong Gierveld and Van Tilburg [86], in which the authors propose a brief 6-item version of the RTLS. This brief 6-item version is commonly used in the literature. However, it is not always clear whether the original or adapted items were used, and sometimes even a mix of original and adapted items has been used (e.g., [87,88]). At the same time, the differences between items are relatively minor; therefore, we think that the different versions are still comparable. 

The scale has been developed in Dutch, for use with (older) adults. Of all studies included in the MASLO database, 8.38% used the RTLS. This scale has mostly been used with older adults (58.69%) and adults (36.62%), and rarely with adolescents (2.35%) and college students (2.35%). The RTLS has been translated in several languages and is used across the world, but mainly in Europe (78.47%), in most cases the Netherlands, where the scale was developed (56.81% of all studies included in the MASLO database using the RTLS were conducted in the Netherlands). The scale has further been used in North America (11.00%), Asia (5.26%), and Australia (5.26%). 

The RTLS was developed as a multidimensional scale that included five subscales. However, only four subscales met the Rasch scale criteria (feelings of severe loneliness, feelings of loneliness connected with specific problem situations such as abandonment, loneliness related to missing companionship, and feelings of belongingness; those are the labels used in the discussion section of the original paper, but throughout the study reporting on the development of the RTLS, different labels were presented). At the same time, the authors also concluded that the results of the analyses performed pointed in the direction of unidimensionality and a methodological artifact reflecting item wording [17]. In the literature, the original four (of five) subscales are very rarely used. In most studies, the RTLS is used as a unidimensional measure. Some studies, however, used the RTLS as a multidimensional measure, distinguishing between emotional and social loneliness. The two factors that reflect emotional and social loneliness include respectively all negatively and positively worded items [86]. Moreover, not all items seem to match the definitions of emotional and social loneliness, questioning the suitability of those two subscales. For example, the emotional loneliness subscale includes the item “I miss having a really close friend”, but also “I miss having people around”. Based on our coding of all 11 RTLS items (see Table 3), 6 items (55%) measure emotional loneliness, and 4 items (36%) measure social loneliness. We do not regard the remaining item as measuring loneliness. Some of the items refer to “friend”, whereas others are more general and refer to “others” or “people”. Regarding the correlation between the emotional and social loneliness subscale, as defined by De Jong Gierveld and Van Tilburg, back-transformed results from the random-effects meta-analysis (*k* = 9) yielded an estimated mean effect size of *r* = 0.47, 95% CI [0.40, 0.53].

#### 3.3.1. Evidence on the Factor Structure of the RTLS

Using Exploratory Factor Analysis, a 1-factor solution was found in a sample of Dutch adults [17]. More frequently, a 2-factor solution has been found, with the two factors reflecting positively and negatively worded items, that is, in a sample of Dutch adults [86], a sample of Dutch older adults [87], and a sample of Canadian adults [56]. In addition, a 3-factor solution was found in a sample of Hebrew older adults [89] and in samples of Turkish-Dutch older adults and Moroccan Dutch older adults [90], and a 4-factor solution was found in samples of Surinamese Creole Dutch, Surinamese Hindustani Dutch, and Dutch older adults [90]. 

Based on confirmatory factor analyses, good fit was found for a 1-factor model in a sample of Belgian adolescents [59]. Evidence for a 1-factor model was further found in Spanish older adults [91,92]. However, in other studies, the 1-factor model showed a poor fit to the data [56,93,94]. Instead, in a sample of Canadian adults, acceptable fit was found for a 2-factor model reflecting item wording, and slightly better fit when one cross-loading was allowed [56]. Better fit for a 2-factor model reflecting item wording was also found in a sample of Polish early adolescents, but fit improved for a bi-factor model where in addition to the two factors reflecting item wording, a general loneliness factor was specified on which all items loaded [94]. Good fit for a 2-factor model was found for a sample of Turkish-Dutch older adults, but not for samples of Moroccan-Dutch, Surinamese-Dutch, and Dutch older adults [90]. Insufficient fit for the 2-factor model was further found for a sample of Polish bilingual university students [93]. In this sample, good fit was found for a bifactor model with two factors reflecting item wording, and one general loneliness factor on which all items could load.

**Table 3:** Coding of Items of the Rasch-Type Loneliness Scale (RTLS).

Item	Emotional Loneliness	Social Loneliness	No Loneliness
I miss having a really close friend	X		
I experience a general sense of emptiness			X
I often feel rejected		X	
I miss having people around		X	
I miss the pleasure of the company of others		X	
I find my circle of friends and acquaintances too limited		X	
There is always someone I can talk to about my day-to-day problems	X		
There are plenty of people that I can lean on when I have problems	X		
There are enough people I feel close to	X		
I can call on my friends whenever I need them	X		
There are many people I can trust completely	X		

#### 3.3.2. Evidence on Measurement Invariance of the RTLS

Several studies have examined measurement invariance, mostly across gender, age, and time. Regarding gender, no invariance could be established in a sample of Dutch older adults [87]. However, in other studies, scalar invariance across gender was established, including a sample of Belgian adolescents [59], Spanish community-dwelling adults [92], and, concerning the 6-item RTLS version, a sample of Brazilian students [95] and Dutch adolescents and adults [96]. Regarding age, scalar invariance has been established in a sample of Canadian adults [56], Spanish community-dwelling adults [92], and, for the 6-item version, a sample of German adults [97] and in a Dutch sample including participants from adolescence until old age [96]. 

Measurement invariance was further established between the Polish and English version in a sample of Polish bilingual students [93]. In a sample of Spanish community-dwelling adults [92], invariance was established across parental status and self-rated health, but not across marital status or living arrangements. Longitudinal measurement invariance has also been examined in several studies. In a sample of Norwegian adults including a 3-item version of the RTLS, metric and partial scalar invariance was established across two measurements that were 5 years apart [98]. In addition, scalar invariance was established across two weeks in a sample of Canadian adults [56], across 1 year in a sample of Polish early adolescents [94], and, for a 6-item version, across 9 years in a sample of Dutch adolescents and adults [96].

#### 3.3.3. Evidence on the Reliability of RTLS Scores

Back-transformed results from the three-level meta-analysis, based on 94 effect sizes, revealed a mean internal consistency reliability coefficient of 0.84, 95% CI [0.83, 0.86]. Results regarding the moderator analyses can be found in Appendix A. The moderator Subscale, including three categories reflecting total loneliness scores, emotional loneliness scores, and social loneliness scores, did not reach significance. The other non-significant moderators were Gender, Country, Translated, and Adaptation. General population and Age did reach significance. Results revealed that reliability estimates were on average somewhat lower for non-clinical samples (mean estimate = 0.83, 95% CI [0.82, 0.85]) than for samples including participants with special education needs or mental health problems (mean estimate = 0.90, 95% CI [0.85, 0.93]). The mean reliability of scores obtained in samples of participants with physical health problems (mean estimate = 0.89, 95% CI [0.83, 0.92]) was not significantly different from the other two categories (i.e., non-clinical samples and samples including participants with special education needs or mental health problems). In addition, reliability estimates were on average somewhat higher in samples of young adults (mean estimate = 0.89, 95% CI [0.86, 0.91]) than in samples of older adults (mean estimate = 0.81, 95% CI [0.78, 0.84]). Mean reliability scores obtained in samples of adolescents (mean estimate = 0.84, 95% CI [0.76, 0.89]) and middle-aged adults (mean estimate = 0.85, 95% [0.82, 0.88]) were not significantly different from the other age categories. The publication bias test did not reveal evidence for bias (*p* = 0.616).

#### 3.3.4. Evidence on Test-Retest Reliability of RTLS Scores

We could not identify any studies that examined the test-retest reliability of RTLS scores obtained maximum one month apart.

### 3.4. The Social and Emotional Loneliness Scale for Adults (SELSA)

The SELSA consists of 37 items with 7 answering categories, ranging from 1 (strongly agree) to 7 (strongly disagree). Common brief versions of the SELSA, the abbreviated SELSA [99] and the SELSA-S [100,101], include 15 items. However, the abbreviated SELSA and the SELSA-S do not contain the same set of 15 items.

The scale has been developed in English, for use with adults. Of all studies included in the MASLO database, 2.79% used this scale. The SELSA has mostly been used with adults (49.30%) and college students (30.99%), and to a lesser extent with adolescents (16.90%) and older adults (2.82%). The SELSA has been translated in several languages and is used across the world, mainly in North America (41.43%) and Europe (37.14%), but also in Asia (11.43%) and Australia (10.00%). 

The SELSA has been developed as a multidimensional scale and includes subscales reflecting social loneliness (14 items) and two domains of emotional loneliness, that is, family loneliness (11 items) and romantic loneliness (12 items). This distinction of subscales according to emotional and social loneliness, however, does not fully correspond to our coding of items (see Table 4). Regarding the Romantic loneliness subscale, we coded seven items (58% of the total number of items of that subscale) as emotional loneliness. We did not regard the other five items (42%) as measuring loneliness. Regarding the Family loneliness subscale, six items (55%) measure emotional loneliness, three items (27%) measure social loneliness, and two items (18%) do not measure loneliness. Regarding the Social loneliness subscale, three items (21%) measure emotional loneliness and eleven items (79%) measure social loneliness. Items in the Romantic loneliness subscale refer to “someone” or a “romantic partner”, items in the Family loneliness subscale refer to “my family”, and items in the Social loneliness subscale refer mostly to “friends”, but also to “people” and “others”. Regarding the correlations among the three subscales, back-transformed results from random-effects meta-analyses yielded an estimated mean effect size of *r* = 0.22, 95% CI [0.16, 0.29] for the association between romantic and family loneliness, an estimated mean effect size of *r* = 0.29, 95% CI [0.23, 0.35] for the association between romantic and social loneliness, and an estimated mean effect size of *r* = 0.38, 95% CI [0.32, 0.43] for the association between family and social loneliness.

#### 3.4.1. Evidence on the Factor Structure of the SELSA

In a sample of Canadian university students, exploratory factor analysis yielded three factors, confirming the proposed factor structure of the SELSA [18]. To our knowledge, no evidence from confirmatory factor analyses is available for the full 37-item version of the SELSA. Regarding the brief 15-item versions of the SELSA, good fit has generally been found for a 3-factor solution corresponding to the proposed factor structure [99,101,102,103,104,105]. 

#### 3.4.2. Evidence on Measurement Invariance of the SELSA

To our knowledge, measurement invariance has not yet been examined for the full 37-item version of the SELSA. Regarding the brief 15-item versions, scalar invariance across gender was found in a sample of Dutch university students [105] and in a sample of Norwegian university students [106]. In this latter sample of Norwegian university students, longitudinal measurement invariance was also tested with results yielding scalar invariance across two measurement waves that were three months apart.

#### 3.4.3. Evidence on the Reliability of SELSA Scores

Back-transformed results from the three-level meta-analysis, based on 108 effect sizes, revealed a mean internal consistency coefficient of 0.85, 95% CI [0.83, 0.87]. Results regarding the moderator analyses can be found in Appendix A. The moderator Subscale, including three categories reflecting social, family, and romantic loneliness, did not reach significance. The other non-significant moderators were General population, Age, Gender, and Country. The moderators Translated and Adaptation did reach significance. Results revealed that reliability estimates were on average somewhat higher in studies that used the English version of the SELSA (mean estimate = 0.87, 95% CI [0.85, 0.89]) than for studies that used a non-English version of the SELSA (mean estimate = 0.81, 95% CI [0.78, 0.85]). In addition, reliability estimates were on average somewhat higher in studies in which the original scale was used (mean estimate = 0.90, 95% CI [0.87, 0.92]) and in studies in which another number of response categories was used (mean estimate = 0.89, 95% CI [0.84, 0.92]), as compared to studies in which a brief version was used (mean estimate = 0.84, 95% CI [0.82, 0.86]) and studies in which versions were used with another number of items and another number of response categories (mean estimate = 0.78, 95% CI [0.70, 0.84]). The publication bias test did not reveal evidence for bias (*p* = 0.736).

#### 3.4.4. Evidence on Test-Retest Reliability of SELSA Scores

One study examined the test-retest reliability of SELSA scores [103]. In this study, Turkish university students filled out a brief 15-item version of the SELSA at two measurement occasions that were two weeks apart. Test-retest reliability coefficients were *r* = 0.88 for social loneliness, *r* = 0.83 for family loneliness, and *r* = 0.91 for romantic loneliness. 

### 3.5. The Differential Loneliness Scale (DLS)

The DLS consists of 60 items with two response categories, that is, T (true) and F (false). There is also a brief 20-item version [107], but it has only rarely been used in the literature. The scale has been developed in English, and two versions were constructed, a student version and a non-student version. Both versions contain a different set of items; according to the authors, there is only 27% overlap in the items between those two versions [19]. However, in the article describing the development of the DLS [19], only the items of the non-student version are presented. This is also the set of items that we based our coding on, and, most likely, this is the version that has been used in the literature. 

Of all studies included in the MASLO database, 0.51% used this scale. The DLS has mostly been used with college students (69.23%), and rarely with adolescents (7.69%), adults (15.38%), and older individuals (7.69%). The large majority of studies including this scale were conducted in North America (72.73%), and rarely in other continents, such as Europe (18.18%) and Asia (9.09%). 

In the original development papers for the DLS, the authors emphasized the subjective discrepancy between actual and desired social relationships, and, thus, developed the DLS as a multidimensional scale. The DLS includes four subscales reflecting loneliness in romantic/sexual relationships (12 items), friendships (22 items), relationships with family (18 items), and relationships with larger groups or the community (8 items). Regarding loneliness in romantic/sexual relationships, we coded 5 items (42% of the total number of items of that subscale) as emotional loneliness, and we did not regard the other 7 items (58%) as measuring loneliness (see Table 5). Regarding loneliness in friendships, we coded 11 items (50%) as emotional loneliness, 2 items (9%) as social loneliness, and 9 items (41%) as not measuring loneliness. Regarding loneliness in relationships with family, we coded 2 items (11%) as emotional loneliness, 1 item (6%) as social loneliness, and 15 items (83%) as not measuring loneliness. Regarding loneliness in relationships with larger groups or the community, we coded 1 item (13%) as emotional loneliness, 6 items (75%) as social loneliness, and 1 item (13%) as not measuring loneliness. Items of the romantic subscale mostly refer to “a romantic relationship” or “partner”, but also to “lover” or “spouse”. Items of the friendship subscale mostly refer to “friends”, but also to “a friend”. Items of the family subscale refer to mostly to “(members of) my family”. Items of the group subscale refer to “people in my community”, but also “group(s)”. Regarding the correlations among the four subscales, back-transformed results from random-effects meta-analyses yielded an estimated mean effect size of *r* = 0.34, 95% CI [0.15, 0.50] for the association between romantic and friendship loneliness, an estimated mean effect size of *r* = 0.34, 95% CI [0.05, 0.57] for the association between romantic and family loneliness, an estimated mean effect size of *r* = 0.31, 95% CI [0.04, 0.54] for the association between romantic and group loneliness, an estimated mean effect size of *r* = 0.36, 95% CI [0.14, 0.54] for the association between friendship and family loneliness, an estimated mean effect size of *r* = 0.58, 95% CI [0.46, 0.67] for the association between friendship and group loneliness, and an estimated mean effect size of *r* = 0.49, 95% CI [0.26, 0.67] for the association between family and group loneliness.

#### 3.5.1. Evidence on the Factor Structure of the DLS

Exploratory factor analyses have been conducted in three studies. In two samples of Canadian university students and adults, respectively, the authors concluded that evidence was found for a 4-factor model [19]. However, the four factors found do not seem to represent the four anticipated types of loneliness. In a sample of German female students, a 3-factor solution was found, reflecting loneliness in relationships with family, in romantic/sexual relationships, and in relationships with friends and larger groups [108]. In a sample of Finnish adults in a mental growth group and adult students of psychology, a 10-factor solution was found [109]. To our knowledge, no evidence from confirmatory factor analysis is available.

#### 3.5.2. Evidence on Measurement Invariance of the DLS

To our knowledge, measurement invariance has not yet been examined for the DLS.

#### 3.5.3. Evidence on the Reliability of DLS Scores

Back-transformed results from the three-level meta-analysis, based on 29 effect sizes, revealed a mean internal consistency of 0.83, 95% CI [0.79, 0.86]. Results regarding the moderator analyses can be found in Appendix A. The moderator Subscale, including four categories reflecting romantic, friends, family, and community loneliness, was significant. The highest mean reliability estimates were found for the subscales reflecting romantic loneliness (mean estimate = 0.89, 95% CI [0.85, 0.92]) and family loneliness (mean estimate = 0.86, 95% CI [0.81, 0.89]). A lower average reliability estimate was found for the subscale reflecting loneliness in friendships (mean estimate = 0.78, 95% CI [0.72, 0.83]). The lowest average reliability estimate was found for the subscale reflecting loneliness in larger groups or the community (mean estimate = 0.61, 95% CI [0.47, 0.72]). The other moderators tested were not significant, that is, Age, Gender, Country, and Translated. The publication bias test did not reveal evidence for bias (*p* = 0.599).

#### 3.5.4. Evidence on Test-Retest Reliability of DLS Scores

One study examined the test-retest reliability of the full 60-item DLS [19]. In a sample of undergraduate university students assessed twice with an interval of one month, test-retest reliabilities were 0.85 and 0.97 for men and women, respectively. In another study, test-retest reliability was examined for scores obtained with a brief 20-item version of the DLS [107]. In a sample of Romanian adults, assessed twice over a period of one month, test-retest coefficients were found of 0.88 for the total score, 0.91 for the subscale on romantic/sexual relationships, 0.75 for the subscale on friendships, 0.89 for the subscale on relationships with family, and 0.48 for the subscale on relationships with larger groups.

### 3.6. The Loneliness and Aloneness Scale for Children and Adolescents (LACA)

The LACA, sometimes also called the “Louvain Loneliness Scale for Children and Adolescents” (LLCA), or, in Dutch, the “Leuvense Eenzaamheidsschaal voor Kinderen en Adolescenten” (LEKA) consists of 48 items, half of which assess loneliness. The other 24 items assess positive and negative attitudes toward aloneness and are not discussed in the present review. The items can be answered on a 4-point scale, ranging from 1 (never) to 4 (often), and, hence, measure the frequency of loneliness experiences. Several brief versions have been used in the literature, but there is no consensus on a particular brief version to be used. 

The scale has been developed in Dutch, for use with older children and adolescents, that is, for the age range of 10–19 years. Of all studies included in the MASLO database, 2.44 used this scale. The scale is mostly used with adolescents (74.19%), but also with children (19.35%) and college students (6.45%). The LACA has been translated into many languages and is used across the world, but mainly in Europe (91.80%). The scale has further been used in North America (1.64%), Asia (4.92%), and South America (1.64%). 

In the original development papers for the LACA, the authors define loneliness in terms of unmet expectations of social relationships. Thus, the LACA was developed as a multidimensional scale, including two subscales reflecting loneliness in relationships with parents (12 items) and loneliness in relationships with peers (12 items). Regarding loneliness in relationships with parents, we coded 3 items (25% of the total number of items of that subscale) as emotional loneliness and 4 items (33%) as social loneliness (see Table 6). The other 5 items (42%), we did not regard as measuring loneliness. Regarding loneliness in relationships with peers, we coded 1 item as emotional loneliness (8%), 9 items (75%) as social loneliness, and 2 items (17%) as not measuring loneliness. Items of the parents subscale refer to “my parents”, except for one items referring to “at home”. Items of the peers subscale refer to “friends”, but also to “others” or “classmates”. Regarding the correlation between the parents- and peer-related loneliness subscale, back-transformed results from the random-effects meta-analysis (*k* = 20) yielded an estimated mean effect size of *r* = 0.22, 95% CI [0.18, 0.26].

#### 3.6.1. Evidence on the Factor Structure of the LACA

In this section, we also consider the two subscales of the LACA that do not measure loneliness (but instead measure attitudes towards aloneness), as those subscales have been part of the exploratory and confirmatory factor analyses conducted. Exploratory factor analyses yielded a 4-factor solution reflecting two loneliness factors and two factors on attitudes towards aloneness, in a sample of Italian adolescents [110] and in a sample of Belgian adolescents [20]. Based on confirmatory factor analyses, in a sample of Belgian and Chinese adolescents in which only peer-related loneliness and the two attitudes scales were included, sufficient model fit was found for a 3-factor model [111]. Good fit for a 4-factor solution was found in two samples of Belgian adolescents [112]. Additionally, when comparing to more parsimonious models, the 4-factor model was found to be superior in Flemish- and French-speaking adolescents [113,114]. In a sample of Italian adolescents [110], three items were deleted based on a reliability analysis (one from the parents- and two from the peer-related loneliness subscale), yielding acceptable model fit for the proposed 4-factor model. Model fit further improved after adding two error correlations. 

#### 3.6.2. Evidence on Measurement Invariance of the LACA

Scalar invariance across gender has been established in several samples of Belgian children and adolescents [59,112,113,114]. Scalar invariance across age has been established in a sample of Belgian early through late adolescents [113] and in a sample of Belgian children, adolescents, and freshmen university students [114]. In a sample of Belgian adolescents, scalar invariance was further established across language, that is, Flemish- and French-speaking adolescents [113]. In a study on Belgian and Chinese adolescents that only included the peer- but not the parent-related loneliness scale, metric and partial scalar invariance across culture was established [111]. In a sample of Belgian and Italian adolescent, metric invariance across culture was established (scalar invariance was not tested; [115]). Longitudinal invariance has been examined in two samples of Belgian adolescents, and scalar invariance was established across three and four waves, respectively, with 1-year intervals [112].

#### 3.6.3. Evidence on the Reliability of LACA Scores

For the LACA, a Reliability Generalization study had already been conducted [116]. Results of this study revealed an estimated mean reliability coefficient of 0.87, 95% CI [0.86, 0.88] for parent-related loneliness (*k* = 77), and 0.87, 95% CI [0.87, 0.88] for peer-related loneliness (*k* = 88). Several moderators were tested as well. For the parent-related loneliness subscale, results of [116] revealed a lower mean reliability coefficient for samples of adolescents as compared to children, and a higher mean reliability coefficient for studies that sampled from multiple cities as compared to studies that sampled from a single city. For the peer-related loneliness subscale, results also revealed a negative effect of age, although in the final model, the mean estimated reliability coefficient was 0.89 for both children and adolescents. Results further indicated a lower estimated mean reliability for studies conducted with Dutch-speaking samples when compared to non-Dutch-speaking samples.

#### 3.6.4. Evidence on Test-Retest Reliability of LACA Scores

To the best of our knowledge, no studies are available that examined the test-retest reliability of LACA scores obtained a maximum of one month apart. However, in the manual of the LACA [117], several test-retest reliabilities were reported. For the peer- and parent-related loneliness subscales, respectively, test-retest reliabilities were 0.74 and 0.70 in a sample of older children with an interval of 2–4 weeks, 0.79 and 0.88 in a sample of adolescents with an interval of 2–4 weeks, and 0.80 and 0.91 in a sample of university students with an interval of 1 week.

### 3.7. The Relational Provisions Loneliness Questionnaire (RPLQ)

The RPLQ consists of 24 items with 5 answering categories, ranging from 1 (no, not at all) to 4 (yes, always), reflecting both frequency and agreement. There is no frequently used brief version available. 

The scale has been developed in English, for use with children. Of all studies included in the MASLO database, 0.24% used this scale. The scale is used with children (66.67%) and adolescents (33.33%). The RPLQ has been translated to some languages and has been used in North America (66.67%), Europe (16.67%), and Australia (16.67%). 

The RPLQ was developed as a multidimensional scale and includes four subscales of seven items each, reflecting peer personal intimacy, family personal intimacy, peer group integration, and family group integration. The former two subscales were developed to reflect emotional loneliness in the peer and family context, respectively, and the latter two subscales were developed to reflect social loneliness in the peer and family context, respectively. Regarding peer personal intimacy, we coded all seven items (100% of the total number of items of that subscale) as emotional loneliness (see Table 7). Regarding family personal intimacy, we also coded all seven items (100%) as emotional loneliness. Regarding peer group integration, we coded all seven items (100%) as social loneliness. Regarding family group integration, we coded six items (86%) as social loneliness and one item (14%) as not measuring loneliness. The items of the peer personal intimacy subscale refer to “someone my age” or “a friend”. The items of the family personal intimacy subscale refer to “someone in my family”. The items of the peer group integration subscale refer to “other children” and “friends”. The items of the family group integration subscale refer to “my family” or “people in my family”. Regarding the correlation between the subscales, back-transformed results from the fixed-effects meta-analysis yielded an estimated mean effect size of *r* = 0.31, 95% CI [−0.13; 0.65] for the association between peer and family personal intimacy and an estimated mean effect size of *r* = 0.40, 95% CI [−0.03, 0.70] for the association between peer and family group integration. Estimated mean effect sizes were *r* = 0.50, 95% CI [0.43, 0.56] for the association between peer personal intimacy and peer group integration, and *r* = 0.77, 95% CI [0.51; 0.90] for the association between family personal intimacy and family group integration. The estimated mean effect size for the association between peer personal intimacy and family group integration was *r* = 0.35, 95% CI [−0.08, 0.68], and *r* = 0.27, 95% CI [−0.17, 0.63] for the association between peer group integration and family personal intimacy.

#### 3.7.1. Evidence on the Factor Structure of the RPLQ

Based on exploratory factor analysis, in a study that included only the peer personal intimacy and peer group integration subscales, a 1-factor model was found in a sample of Norwegian children with physical, intellectual, or multiple disabilities [118]. Based on confirmatory factor analysis, in a study that also only included the two peer-related subscales, good fit was found for a 2-factor model in a sample of Belgian adolescents [59]. In a study including all four subscales, good fit was found for a 4-factor model in a sample of Portuguese adolescents [119].

#### 3.7.2. Evidence on Measurement Invariance of the RPLQ

Scalar invariance across gender was established in a study of Belgian adolescents including the two peer subscales [59] and in a study of Portuguese adolescents including all four subscales [119]. In this latter study on Portuguese adolescents, aged between 11 and 17 years old, scalar invariance across age was also established.

#### 3.7.3. Evidence on the Reliability of RPLQ Scores

Back-transformed results from the three-level meta-analysis, based on 12 effect sizes, revealed a mean internal consistency of 0.84, 95% CI [0.71, 0.91]. Results regarding the moderator analyses can be found in Appendix A. Due to insufficient data, we did not examine the moderator Subscale. We do regard an estimated mean reliability per subscale as informative, and hence report the results here, but note that the estimates are based on only very few effect sizes. For the peer personal intimacy subscale (*k* = 3), the estimated mean reliability = 0.77, 95% CI [0.57, 0.88], for family personal intimacy (*k* = 1) the estimated mean reliability = 0.78, 95% CI [0.57, 0.89], for peer group integration (*k* = 3) the estimated mean reliability = 0.79, 95% CI [0.60, 0.89], and for family group integration (*k* = 1) the estimated mean reliability = 0.81, 95% [0.62, 0.90]. Some studies also combined the two peer and the two family subscales, yielding an estimated mean reliability of 0.90, 95% CI [0.75, 0.96] for the combined two peer subscales (*k* = 2) and an estimated mean reliability of 0.93, 95% CI [0.83, 0.97] for the combined two family subscales (*k* = 2). The only moderator that could be tested was Gender, which was significant, with results suggesting that reliability on average became lower in samples with higher percentages of male respondents. The publication bias test did not reveal evidence for bias (*p* = 0.072).

#### 3.7.4. Evidence on Test-Retest Reliability of RPLQ Scores

To the best of our knowledge, no studies are available that examined the test-retest reliability of RTLS scores obtained maximum one month apart.

### 3.8. The Peer Network and Dyadic Loneliness Scale (PNDLS)

The PNDLS consists of 16 items with 4 answering categories, in “Harter’s format”. Specifically, participants are presented with pairs of sentences describing children. For each pair, they are then asked to select the sentence describing the child that is most like them. Next, participants indicate whether the selected description *is sort of true* or *really true* for them. In essence, scores range from 1 (very low loneliness) to 4 (very high loneliness). There is no frequently used brief version available.

The scale has been developed in English, for use with children. Of all studies included in the MASLO database, 0.51% used this scale. The scale is used with children (61.54%) and adolescents (38.46%). The PNDLS has been translated is some languages and has been used in North-America (53.85%), Europe (23.08%), and Asia (23.08%). 

The PNDLS has been developed as a multidimensional scale, including a peer dyadic and peer network loneliness subscale, of 8 items each, intended to reflect emotional and social loneliness in the peer context, respectively. Later, two additional subscales were developed, reflecting family dyadic and family loneliness. Those family subscales have been used in the literature, but only rarely, and the items have not yet been published. Regarding the peer dyadic loneliness subscale, we coded 6 items (75% of the total number of items of that subscale) as emotional loneliness, 1 item (13%) as social loneliness, and 1 item (13%) as not measuring loneliness (see Table 8). Regarding the peer network loneliness subscale, we coded 7 items (88%) as social loneliness, and 1 item (13%) as not measuring loneliness. The items of the peer dyadic subscale refer to “a friend” of “someone their age”. The items of the peer network subscale refer mostly to “other kids”. Regarding the correlation between the peer dyadic and network loneliness scales, back-transformed results from the fixed-effects meta-analysis (*k* = 4) yielded an estimated mean effect size of *r* = 0.62, 95% CI [0.56; 0.67].

#### 3.8.1. Evidence on the Factor Structure of the PNDLS

Based on exploratory factor analysis, a 2-factor solution was found in a sample of US children [22]. Based on confirmatory factor analysis, good fit was found for a 2-factor model in a sample of Belgian adolescents [59], and in a sample of Finnish adolescents using a brief version of the PNDLS [120].

#### 3.8.2. Evidence on Measurement Invariance of the PNDLS

Scalar invariance across gender has been established in a sample of Belgian adolescents [59]. In a sample of Finnish adolescents, using a brief version of the PNDLS, longitudinal metric invariance was established across measurement waves with 6-months intervals (scalar invariance was not tested; [120]).

#### 3.8.3. Evidence on the Reliability of PNDLS Scores

Back-transformed results from the three-level meta-analysis, based on 16 effect sizes, revealed a mean internal consistency reliability coefficient of 0.84, 95% CI [0.82, 0.86]. Results regarding the moderator analyses can be found in Appendix A. The moderator Subscale, including the subscales peer network and dyadic loneliness, did not reach significance. The other non-significant moderators were Gender, Country, Translated, and Adaptation. The moderator Age did reach significance, showing that the reliability estimates were on average somewhat lower in samples of children (mean estimate = 0.82, 95% CI [0.79, 0.84]) than in samples of adolescents (mean estimate = 0.86, 95% CI [0.84, 0.87]). The publication bias test did not reveal evidence for bias (*p* = 0.093).

#### 3.8.4. Evidence on Test-Retest Reliability of PNDLS Scores

To the best of our knowledge, no studies are available that examined the test-retest reliability of PNDLS scores obtained maximum one month apart.

### 3.9. Associations among the Loneliness Measures

Table 9 shows the back-transformed results from the meta-analyses on the association between the different loneliness measures. As can be seen in this table, information for several of the potential correlations is missing. For example, for the RTLS, only the correlation with the UCLA is available. In general, little evidence is available regarding the associations among measures that are usually used with adults (UCLA, RTLS, SELSA, and DLS), and the scales for children and adolescents (CLS, LACA, RPLQ, and PNDLS).

Rather high correlations were found for the family subscales of the SELSA and the DLS, and for the social subscale of the SELSA and the friendship subscale of the DLS. No information was available for the correlation between the romantic subscales of the SELSA and the DLS. Similar patterns emerged for the correlations among the subscales of the LACA and the RPLQ, that is, strong correlations were found between the parent and peers subscales of the LACA, respectively, and the family and peer subscales of the RPLQ, respectively. No correlations with other scales were available for the PNDLS. 

Both the UCLA and the CLS have been developed as unidimensional scales, assessing “general loneliness”, which would suggest that they measure different types of loneliness in a balanced way. Looking at the correlations of those two scales with the other measures, the UCLA correlates highly with the social subscale of the SELSA and with the friendships subscale of the DLS. Many items of the social subscale of the SELSA refer to “friends”, and most items measure social loneliness. Most of the friendship subscale of the DLS also refers to “friends”, but mainly measures emotional loneliness. On the contrary, the CLS is strongly correlated with the peer subscale of the LACA and the peer group subscale of the RPLQ, which both measure social loneliness in the peer context. This pattern of correlations is in line with our coding, suggesting that the UCLA measures both emotional and social loneliness, whereas the CLS measures mostly social loneliness.

**Table 9:** Results of the Separate Meta-Analyses on the Correlations Among Loneliness (Sub)Scales.

		UCLA		CLS		RTLS					SELSA							DLS								LACA				RPLQ							PNDLS	
						Total		Emotional		Social	Total	Romantic		Family		Social		Total	Romantic		Family		Friendships		Community	Parents		Peers		Family Personal		Peer Personal		Family Group		Peer Group	Peer Dyadic	
CLS				─																																		
RTLS	Total	0.78	[0.72; 0.82]			─																																
		*k* = 3																																				
	Emotional					0.79	[0.77; 0.80]	─																														
						*k* = 3																																
	Social					0.85	[0.84; 0.86]	0.47	[0.40; 0.53]	─																												
						*k* = 3		*k* = 9																														
SELSA	Total										─																											
	Romantic	0.31	[0.25; 0.38]									─																										
		*k* = 12																																				
	Family	0.34	[0.23; 0.45]									0.22	[0.16; 0.29]	─																								
		*k* = 8										*k* =18																										
	Social	0.58	[0.47; 0.67]									0.29	[0.23; 0.35]	0.38	[0.32; 0.43]	─																						
		*k* = 13										*k* = 22		*k* = 20																								
DLS	Total	0.68	[0.63; 0.73]															─																				
		*k* = 4																																				
	Romantic	0.33	[−0.11; 0.66]																─																			
		*k* = 2																																				
	Family	0.32	[0.24; 0.39]											0.78	[0.53; 0.91]	0.25	[−0.21; 0.62]		0.34	[0.05; 0.57]	─																	
		*k* = 4												*k* = 2		*k* = 2			*k* = 5																			
	Friendships	0.65	[0.60; 0.70]											0.31	[−0.15; 0.65]	0.68	[0.34; 0.86]		0.34	[0.15; 0.50]	0.36	[0.14; 0.54]	─															
		*k* = 4												*k* = 2		*k* = 2			*k* = 5		*k* = 6																	
	Community																		0.31	[0.04; 0.54]	0.49	[0.26; 0.67]	0.58	[0.46; 0.67]	─													
																			*k* = 5		*k* = 5		*k* = 5															
LACA	Parents			0.35	[0.25; 0.44]																					─												
				*k* = 5																																		
	Peers			0.74	[0.68; 0.79]																					0.22	[0.18; 0.26]	─										
				*k* = 5																						*k* = 20												
RPLQ	Family personal			0.24	[−0.20; 0.61]																					0.62	[0.27; 0.83]	0.15	[−0.30; 0.54]	─								
				*k* = 2																						*k* = 2		*k* = 2										
	Peer personal			0.46	[0.05; 0.74]																					0.23	[−0.22; 0.59]	0.39	[−0.05; 0.70]	0.31	[−0.13; 0.65]	─						
				*k* = 2																						*k* = 2		*k* = 2		*k* = 2								
	Family group			0.33	[−0.11; 0.66]																					0.69	[0.37; 0.86]	0.22	[−0.23; 0.59]	0.77	[0.51; 0.90]	0.35	[−0.08; 0.68]	─				
				*k* = 2																						*k* = 2		*k* = 2		*k* = 2		*k* = 2						
	Peer group			0.69	[0.37; 0.86]																					0.29	[−0.15; 0.64]	0.59	[0.21; 0.81]	0.27	[−0.17; 0.63]	0.50	[0.43; 0.56]	0.40	[−0.03; 0.70]	─		
				*k* = 2																						*k* = 2		*k* = 2		*k* = 2		*k* = 3		*k* = 2				
PNDLS	Peer dyadic																																				─	
	Peer network																																				0.62	[0.56; 0.67]
																																					*k* = 4	

*Note.* Results represent the estimated mean effect sizes with 95% confidence intervals. *k* = number of effect sizes.

## 4. Discussion

In the present review paper, we focused on the eight most commonly used loneliness questionnaires: the UCLA, CLS, RTLS, SELSA, DLS, LACA, RPLQ, and PNDLS. We discussed the distinct loneliness type(s) they measure, and we reviewed the evidence on the psychometric properties of those scales, focusing on factor structure, internal consistency, test-retest reliability, and measurement invariance. Moreover, we clarified how the different scales relate to each other. 

Strikingly, all loneliness scales contained items that fail to reflect the subjective nature of loneliness. That is, loneliness arises when people *perceive* a discrepancy between their actual and desired social relationships. For example, the item “I have lots of friends” (CLS) would better fit the formal definition of loneliness if it explicitly asks whether people think they have as many friends as they would like to, or whether they feel their friendships are of high enough quality. Similarly, all loneliness scales contain items that are related or predictive of loneliness to some extent, but do not measure loneliness per se. Examples are “I am an outgoing person” (UCLA), “It’s easy for me to make new friends at school” (CLS), “I find it easy to express feelings of affection toward members of my family” (DLS), and “I find it hard to talk to my parents” (LACA). Thus, many scales contain items that conceivably are related to or may predict loneliness, but do not actually reflect loneliness per se. 

Even though all scales included items that do not actually measure loneliness, the extent to which such items were included greatly differed across the scales. There were several scales of which 40% or more of the items did not measure loneliness. These scales were, for use with children and adolescents, the CLS (50% of the items do not measure loneliness) and the parent-related loneliness scale of the LACA (42%), and, for use with adults, the romantic loneliness scale of the SELSA (42%) and three of the four DLS subscales, that is, the subscales reflecting loneliness in friendships (41%), romantic relationship (42%), and the family (83%). The other loneliness measures performed better in this regard, as we coded none or only one or two of the items as not assessing loneliness. Specifically, the UCLA includes two items that do not measure loneliness (10% of the total number of items), the peer-related subscale of the LACA includes two items (17%), the group loneliness subscale of the DLS includes one item (13%), the RTLS includes one item (9%), and for the SELSA, the family loneliness subscale includes two items not measuring loneliness (18%) and the social loneliness subscale includes no such items. Strikingly, the two loneliness measures that were developed for use with children but that have so far been used rather rarely in the literature performed very well in this regard. Specifically, for three of the four subscales of the RPLQ, none of the items were coded as not measuring loneliness, and for the fourth subscale (i.e., family group integration), only one item (14%) was coded as such. For both PNDLS subscales (i.e., peer network and peer dyadic loneliness), only one item per subscale (13%) was coded as not measuring loneliness.

### 4.1. Which Loneliness Types Are Measured?

The CLS, UCLA, and RTLS are generally used as unidimensional measures assessing “general loneliness”. Correspondingly, those three measures include both emotional and social loneliness items; however, they do so to a different extent. Specifically, most items of the CLS assess social loneliness as compared to emotional loneliness (5 vs. 2 items). The UCLA also contains more social than emotional loneliness items (11 vs. 7 items), but for the RTLS it is the other way around (4 social vs. 6 emotional loneliness items). Neither UCLA nor RTLS refer to specific relationships, whereas the items of the CLS refer to peers or friends and focus on the school context. Hence, even though the CLS, UCLA, and RTLS are all used to measure “general loneliness”, they capture different aspects of loneliness to a different extent. 

The other scales, that is, the SELSA, DLS, LACA, RPLQ, and PNDLS, all contain multiple subscales to reflect different types of loneliness. The SELSA measures loneliness in romantic relationships (7 emotional loneliness items), in the family (6 emotional and 3 social loneliness items), and in social relationships, with items referring to “friends” and “people” (3 emotional and 11 social loneliness items). The DLS also contains a subscale intended to measure loneliness in romantic relationships (5 emotional loneliness items), and in the family (2 emotional and 1 social loneliness item). Furthermore, the DLS contains a subscale on loneliness in friendships, but where the friends (“social”) subscale of the SELSA included mostly social loneliness items, the friends subscale of the DLS included mostly emotional loneliness items (11 emotional and 2 social loneliness items). The DLS subscale referring to “people in my community” and “groups” included mostly social loneliness items (6 social and 1 emotional loneliness item). 

Where the SELSA and DLS, both generally used with adults, referred to “family” relationships, the LACA, generally used with adolescents, included a subscale referring to loneliness in relation to parents, with 3 emotional and 4 social loneliness items. The LACA further included a subscale on loneliness in relation to peers (mostly “friends”), essentially measuring social loneliness (1 emotional and 9 social loneliness items). So, although the SELSA, DLS, and LACA all include a subscale measuring loneliness in friendships, they have a different focus concerning emotional vs. social loneliness in this friendship context. Both the RPLQ and PNDLS made this distinction between emotional and social loneliness in the friendship context clear by explicitly developing separate subscales for both types (labeled as peer personal intimacy and peer group integration for the RPLQ and peer dyadic and peer network for the PNDLS. For the family context, the RPLQ further explicitly included two separate subscales to measure emotional and social loneliness (labeled as family personal intimacy and family group integration).

### 4.2. Factor Structure, Internal Consistency, Test-Retest Reliability, and Measurement Invariance

Regarding the psychometric properties, for all scales, at least some evidence was available on their factor structure. However, for the scales that are generally used as unidimensional (i.e., the CLS, UCLA, and RTLS), no consensus has been reached about the factor structure, and different factor solutions have been proposed in the literature. Moreover, there is an ongoing debate in the literature regarding all of those three scales; there are questions about whether the factors represent substantive factors that assess distinct loneliness types or whether it is more of a methodological artifact, where the factors merely represent item wording. That is, positively phrased and negatively phrased items tend to cluster together. 

For the other scales (i.e., the SELSA, DLS, LACA, RPLQ, and PNDLS), which include several subscales, evidence on the factor structure was generally in line with the proposed factor structure, except for the DLS. The factor structure of the DLS has not yet been established, especially concerning the friendship and group subscales, which could not consistently be distinguished. Moreover, factorial evidence for the SELSA and LACA needs to be extended; as for the SELSA, evidence is only available for a brief (and not the full) version. For the LACA, the factor solutions that corresponded with the proposed factor structure were mostly based on parcels (i.e., a sum or average score of 2 or more items; [121]) rather than items. Factor analyses including the items usually led to the deletion of items or the addition of error correlations. Regarding the RPLQ and PNDLS, psychometric evidence is still largely lacking. Therefore, although the factorial evidence seems promising, replication of those findings is highly recommended. 

Regarding the reliability of scale scores, internal consistency has been found to be good for (almost) all (sub)scales, except for the DLS subscale measuring loneliness in larger groups. Regarding test-retest reliability, evidence was largely lacking. For the UCLA, CLS, and LACA, the results seemed promising. For the DLS, results for the total scale seemed promising, but were rather low for the subscale measuring loneliness in larger groups (brief version). For the SELSA, only evidence for a brief version was available, and for the other scales, that is, the RTLS, RPLQ, and PNDLS, no evidence was available. 

Similarly, although increasingly being tested, measurement invariance is currently still largely understudied. Among the many groups for which measurement invariance can be established, gender and age were the most frequently examined ones. In addition, longitudinal invariance also has received some attention in the literature. Most evidence on measurement invariance is available for the UCLA, and, even though rather mixed, results seemed promising. At the same time, however, replication and extension of the available evidence is highly needed. Measurement invariance across age, for example, has only been tested for adult samples, and longitudinal invariance was tested in only one study (in which measurement invariance could not be established). For the CLS, RTLS, and LACA, evidence on measurement invariance, including invariance across gender, age, and time, is also available with rather promising results. For the RPLQ and PNDLS, psychometric evidence is again relatively scarce, but the available evidence in this regard was promising, as measurement invariance was examined and established across gender and age for the RPLQ and across gender and time for the PNDLS. No evidence on measurement invariance was available for the full version of the SELSA, but for a brief version, measurement invariance across gender and time could be established. For the DLS, measurement invariance has not yet been examined.

### 4.3. Selecting a Loneliness Scale for Research Purposes

Several questionnaires have been developed to assess loneliness, each with their own unique characteristics, and their own strengths and weaknesses. Hence, there is no single “best choice”; the best choice depends on the research purpose. Therefore, we encourage scholars to carefully consider what type of loneliness (i.e., emotional vs. social loneliness) fit their theoretical model, and to carefully consider what relationship context their theoretical model of loneliness applies to. Indeed, intercorrelations between various loneliness measures indicate that not all measures are comparable when it comes to the type of loneliness they measure, and correlations between measures of loneliness referring to different relationship contexts only correlate moderately.

#### 4.3.1. Measuring Loneliness among Children and Adolescents

To measure loneliness among children and adolescents, the CLS, LACA, RPLQ, and PNDLS can be used. For children, currently the most often used scale is the CLS. However, 50% of the items of the CLS do not measure loneliness. In addition, it is important to realize that this scale measures loneliness in the peer context, and that the items that do measure loneliness focus more on social than on emotional loneliness. Moreover, even though the CLS is usually used as a unidimensional measure, consensus on the best fitting factor structure is still lacking. 

To measure emotional and social loneliness, that is, to distinguish between both types or to measure general loneliness by combining those types, the RPLQ and PNDLS seem to be better choices. Both scales have been used less often, and, correspondingly, evidence on their psychometric characteristics is still relatively scarce, and future work is needed in this regard. Nevertheless, the psychometric studies that are available are promising. To measure (emotional and social) loneliness in the family context, the RPLQ can be used, and to measure (emotional and social) loneliness in the peer context, both the RPLQ and the PNDLS can be used. Both peer subscales of the PNDLS contain one item that does not measure loneliness, which is not the case for the peer subscales of the RPLQ, but the mean estimated reliability scores were somewhat higher for the PNDLS subscales as compared to the RPLQ subscales. Further, the PNDLS uses answer categories in “Harter’s format”, which might be difficult to fill in for some children. This has been solved in previous work [122] by adapting the items so that a Likert-type answer scale could be used. Yet, psychometric research on the quality of this answer scale is largely absent. 

For adolescents, the LACA could also be used, which includes subscales on both the peer and parent context. Regarding the peer subscale of the LACA, the vast majority of items measure social loneliness. Hence, to measure emotional loneliness (or general loneliness, including both types of loneliness) in the peer context for adolescents, currently, no suitable measure is available. In previous work [59], a solution was proposed by adapting the items of the peer subscales of the RPLQ and PNDLS so that they do not refer to “kids” or “children” anymore, but to “youth” instead. 

Regarding the parent subscale of the LACA, 42% of the items do not measure loneliness, and the other items measure both emotional and social loneliness (with 3 and 4 items, respectively). Hence, if the aim is to measure loneliness in the family context, the family subscales of the RPLQ seem to be the better choice. However, for some research purposes, it might also be relevant to assess loneliness in more specific relationships within the family, such as a sibling or parent. The LACA does focus on parents, instead of the broader family, but for some studies it still might be more relevant to focus on a specific parent. Also, it might be difficult to answer questions such as “I feel I have very strong ties with my parents” (LACA) for both parents simultaneously. At this moment, no scale exists to measure loneliness in specific relationships within the family. 

To measure loneliness in romantic relationships, there is currently no scale available that can be used for adolescents, even though romantic relationships are a significant part of adolescents’ social world [123] and have been shown to be linked directly to experiences of loneliness [124]. In fact, during adolescence, romantic relationships are both normative and salient, with about half of 15-year-olds and almost three quarters of 18-years-olds being involved in a romantic relationship [125]. Thus, the development of such a scale seems warranted.

#### 4.3.2. Measuring Loneliness among Adults

To measure loneliness among adults, the UCLA, RTLS, SELSA, and DLS can be used. Currently, the most often used scale is the UCLA. In most of those studies, the UCLA is used as a unidimensional measure, assessing general loneliness. The UCLA includes more items measuring social than emotional loneliness (i.e., 11 and 7 items, respectively), but does cover both aspects, making it reasonable to use it as a general loneliness measure (although a better balance between the two might be desirable). There is, however, still much debate regarding the factor structure of the UCLA. In several studies, the items of the UCLA were divided in separate factors to reflect different types of loneliness, but those factors have usually not been replicated in other work. Hence, more work in this regard is needed, and, at this moment, we do not recommend the use of subscales for the UCLA. This also means that if the research aim is to distinguish between different types of loneliness, the UCLA would not be the best choice. The same holds for the RTLS. This scale also measures both emotional and social loneliness, but we do not recommend using the emotional and social subscales as proposed in [86], as not all items match the definition of the loneliness types. Moreover, because the division between emotional and social loneliness in this scale matches the wording of the items (i.e., only positively worded items for emotional loneliness and only negatively worded items for social loneliness), it is unclear whether reported factor structures are due to wording or to content of items.

When specifically focusing on older adults, in almost all studies, either the UCLA or the RTLS was used to measure loneliness. As both scales can be used to assess general loneliness, it is yet unknown whether (relation-specific) types of loneliness can be reliably and validly measured in this population. Measurement invariance across age (middle-aged and older adults) has been examined for the UCLA and RTLS, but evidence is still rather scarce, and findings have been mixed.

To measure loneliness in romantic, family, or friend relationships, both the SELSA and DLS contain subscales in that regard. Regarding romantic loneliness, however, we coded most items of both SELSA and DLS subscales as not measuring loneliness. Regarding family relationships, most items of the DLS subscale were regarded as not measuring loneliness. The family subscale of the SELSA is a better choice in this regard; however, note that most items measure emotional rather than social loneliness. Moreover, most items of the SELSA refer to “my family”, whereas for some research purposes, it might be more suitable to focus on a more specific relationship within the family. Hence, future work is needed to develop scales measuring loneliness in relationships with a romantic partner and specific family members. 

Regarding relationships with friends, again, the DLS subscales contains many items that do not seem to be appropriate indicators of loneliness. Most of the other items measure emotional, rather than social, loneliness. Most of the items of the social subscale of the SELSA refer to friendships, with most of them assessing social, rather than emotional, loneliness. In addition, previous work has proposed that loneliness can be experienced within broader groups, such as the community one lives in (also referred to as “collective loneliness”; [23]). This concept is not often used in the literature, and a clear definition is still lacking, but the group subscale of the DLS seems to measure this type of loneliness, at least to some extent. Most of the items of this subscale measure social loneliness. However, the internal consistency of this subscale was found to be low. Moreover, in factor analyses, the friendship and group subscales could not consistently be distinguished. We are therefore hesitant to recommend using this scale to measure collective loneliness.

### 4.4. Limitations

The present review offers a thorough discussion of many loneliness measures available for children, adolescents, and adults. However, there are limitations. The first limitation concerns the MASLO database. This database contains over 2300 studies using (at least) one of the eight loneliness questionnaires, published until 2016. Hence, the most recently published articles are not included in the present review. Still, the different reviews and meta-analyses were based on a substantial set of studies, and it is unlikely that findings (e.g., the reliability estimates for the different scales) would drastically change, given the overall narrow confidence intervals found in the present analysis. 

Another limitation concerns the conceptualization of loneliness. Regarding the general definition of loneliness, most research uses the definition as presented in the current paper. However, there is also research that challenges this standard definition (for example, see [126,127,128], this special issue), which might lead to different theories and a different conceptualization of loneliness, and, in turn, might lead to different measurement instruments. In addition, specifically for the conceptualization of emotional and social loneliness, we operated on a particular definition to code the items of the different scales accordingly. Those definitions were based on a review of previously published work and on extensive discussions among the authors of the present study. Still, although the definitions reflect a considerately reached consensus, other researchers might have arrived at different definitions, and hence, other estimates of percentages of items reflecting respectively emotional and social loneliness. In addition, other types of loneliness have been distinguished in the literature, such as collective loneliness [25] and existential loneliness [129]. However, both types have received far less attention, and additional research is needed to clarify their conceptualization. Moreover, although the items in many subscales may not measure loneliness as it is generally defined by scholars, specifically by capturing discrepancies between desired and actual social relationships, they do pertain to socially healthy social behaviors and relationships, and if that is what scholars intend to measure, they may still be suitable.

## 5. Conclusions

Different scales have been developed to measure loneliness. However, evidence of their psychometric properties is still relatively scarce, especially concerning test-retest reliability and measurement invariance. Most strikingly, although the psychometric evidence that is available is generally reassuring, many items are still not appropriate indicators of loneliness. Surprisingly, for many (sub)scales, about half of the items do not measure loneliness as it is commonly defined. As measurement is the foundation on which our future efforts, such as identifying risk factors or developing or evaluating intervention programs, are built, it is crucial to work with the best measures possible. We need instruments that measure what we intend to measure, and that, preferably, do so across different contexts and populations. Important steps forward have been accomplished, but there is still some way to go.

## Figures and Tables

**Table 2:** Coding of Items of the Children’s Loneliness Scale (CLS).

Item	Emotional Loneliness	Social Loneliness	No Loneliness
It’s easy for me to make new friends at school			X
I have nobody to talk to		X	
I’m good at working with other children			X
It’s hard for me to make friends			X
I have lots of friends			X
I feel alone		X	
I can find a friend when I need one	X		
It’s hard to get other kids to like me			X
I don’t have anyone to play with		X	
I get along with other kids			X
I feel left out of things		X	
There’s nobody I can go to when I need help	X		
I don’t get along with other children			X
I’m lonely			
I am well-liked by the kids in my class			X
I don’t have any friends		X	

**Table 4:** Coding of Items of the Social and Emotional Loneliness Scale for Adults (SELSA).

Item	Emotional Loneliness	Social Loneliness	No Loneliness
*Romantic loneliness*			
I am an important part of someone else’s life			X
I have a romantic partner with whom I share my most intimate thoughts and feelings	X		
There is someone who wants to share their life with me			X
I have a romantic or marital partner who gives me the support and encouragement I need	X		
I have an unmet need for a close romantic relationship	X		
I wish I could tell someone who I am in love with, that I love them			X
I find myself wishing for someone with whom to share my life	X		
I’m in love with someone who is in love with me			X
I wish I had a more satisfying romantic relationship	X		
I have someone who fulfills my needs for intimacy	X		
I have someone who fulfills my emotional needs	X		
I have a romantic partner to whose happiness I contribute			X
*Family loneliness*			
I feel alone when I’m with my family		X	
No one in my family really cares about me	X		
There is no one in my family I can depend upon for support and encouragement, but I wish there were	X		
I really care about my family			X
I really belong in my family		X	
I wish my family was more concerned about my welfare	X		
I feel part of my family		X	
My family really cares about me	X		
There is no one in my family I feel close to, but I wish there were	X		
My family is important to me			X
I feel close to my family	X		
*Social loneliness*			
What’s important to me doesn’t seem important to the people I know		X	
I don’t have a friend(s) who shares my views, but I wish I did		X	
I feel part of a group of friends		X	
My friends understand my motives and reasoning		X	
I feel ‘in tune’ with others		X	
I have a lot in common with others		X	
I have friends that I can turn to for information		X	
I like the people I hang out with		X	
I can depend upon my friends for help	X		
I have friends to whom I can talk about the pressures in my life	X		
I don’t have a friend(s) who understands me, but I wish I did	X		
I do not feel satisfied with the friends that I have		X	
I have a friend(s) with whom I can share my views		X	
I’m not part of a group of friends and I wish I were		X	

**Table 5:** Coding of Items of the Differential Loneliness Scale (DLS).

Item	Emotional Loneliness	Social Loneliness	No Loneliness
*Loneliness in romantic/sexual relationships*			
At this time, I do not have a romantic relationship that means a great deal to me			X
I am now involved in a romantic or marital relationship where both of us make a genuine effort at cooperation			X
I find it difficult to tell anyone that I love him or her			X
I am an important part of the emotional and physical well-being of my lover or spouse			X
I have a lover or spouse who fulfills many of my emotional needs.	X		
Right now, I don’t have true compatibility in a romantic or marital relationship	X		
My romantic or marital partner gives me much support and encouragement	X		
People who say they are in love with me are usually only trying to rationalize using me for their own purposes			X
I don’t have any one special love relationship in which I feel really understood	X		
I have an active love life			X
I tend to get along well with partners in romantic relationships			X
I seldom get the emotional security I need from a romantic or sexual relationship	X		
*Loneliness in friendships*			
Members of my family enjoy meeting my friends			X
I usually wait for a friend to call me up and invite me out before making plans to go anywhere			X
Most of my friends understand my motives and reasoning	X		
I have at least one good friend of the same sex		X	
Some of my friends will stand by me in almost any difficulty	X		
My trying to have friends and to be liked seldom succeeds the way I would like it to			X
I don’t have many friends in the city where I live			X
I don’t feel that I can turn to my friends living around me for help when I need it	X		
My friends are generally interested in what I am doing, although not to the point of being nosy		X	
I allow myself to become close to my friends			X
Few of my friends understand me the way I want to be understood	X		
A lot of my friendships ultimately turn out to be pretty disappointing	X		
I often feel resentful about certain actions of my friends			X
In my relationships, I am generally able to express both positive and negative feelings			X
I get plenty of help and support from friends	X		
I have few friends with whom I can talk openly	X		
I have few friends that I can depend on to fulfill their end of mutual commitments	X		
I have at least one real friend	X		
I have moved around so much that I find it difficult to maintain lasting friendships			X
I find it difficult to invite a friend to do something with me			X
My friends don’t seem to stay interested in me for long	X		
Most of my friends are genuinely concerned about my welfare	X		
*Loneliness in relationships with family*			
I find it easy to express feelings of affection toward members of my family			X
I don’t get along very well with my family			X
I often become shy and retiring in the company of relatives			X
I spend time talking individually with each member of my family			X
I don’t think that anyone in my family really understands me	X		
My relatives are generally too busy with their concerns to bother about my problems			X
Members of my family give me the kind of support that I need	X		
I am not very open with members of my family			X
I am embarrassed about the way my family behaves			X
I have a good relationship with most members of my immediate family			X
I seem to have little to say to members of my family			X
I really feel that I belong to a family		X	
My family is quite critical of me			X
Generally I feel that members of my family acknowledge my strengths and positive qualities			X
Members of my family are relaxed and easy-going with each other			X
I have little contact with members of my family			X
As much as possible, I avoid members of my family			X
My family usually values my opinion when a family decision is to be made			X
*Loneliness in relationships with larger groups or the community*			
I can’t depend on getting moral, or financial support from any group or organization in a time of trouble		X	
Most everyone around me is a stranger		X	
People in my community aren’t really interested in what I think or feel		X	
I work well with others in a group			X
No one in the community where I live cares much about me		X	
I don’t get much satisfaction from the groups I attend		X	
I don’t have any neighbours who would help me out in a time of need	X		
There are people in my community who understand my views and beliefs		X	

**Table 6:** Coding of Items of the Loneliness and Aloneness Scale for Children and Adolescents (LACA).

Item	Emotional Loneliness	Social Loneliness	No Loneliness
*Loneliness in relationships with parents*			
I feel I have very strong ties with my parents	X		
My parents make time to pay attention to me		X	
I feel left out by my parents		X	
I find consolation with my parents	X		
I find it hard to talk to my parents			X
I can get along with my parents very well			X
My parents are ready to listen to me or to help me	X		
I have the feeling that my parents and I belong together		X	
My parents share my interests			X
My parents show real interest in me		X	
I doubt whether my parents love me after all			X
At home I feel at ease			X
*Loneliness in relationships with peers*			
I think I have fewer friends than others			X
I feel isolated from other people		X	
I feel excluded by my classmates		X	
I want to be better integrated in the class group		X	
Making friends is hard for me			X
I am afraid that others won’t let me join in		X	
I feel alone at school		X	
I think there is no single friend to whom I can tell everything	X		
I feel abandoned by my friends		X	
I feel left out by my friends		X	
I feel sad because nobody wants to join in with me		X	
I feel sad because I have no friends		X	

**Table 7:** Coding of Items of the Relational Provisions Loneliness Questionnaire (RPLQ).

Item	Emotional Loneliness	Social Loneliness	No Loneliness
*Peer personal intimacy*			
There is someone my age I can turn to	X		
There is someone my age I could go to if I were feeling down	X		
I have at least one really good friend I can talk to when something is bothering me	X		
I have a friend who is really interested in hearing about my private thoughts and feelings	X		
I have a friend I can tell everything to	X		
There is somebody my age who really understand me	X		
There is a friend I feel close to	X		
*Family personal intimacy*			
There is someone in my family I can turn to	X		
There is someone in my family I could go to if I were feeling down	X		
I have at least one person in my family I can talk to when something is bothering me	X		
I have someone in my family who is really interested in hearing about my private thoughts and feelings	X		
I have someone in my family I can tell everything to	X		
There is someone in my family who really understands me	X		
There is someone in my family I feel close to	X		
*Peer group integration*			
I feel part of a group of friends that does things together		X	
I have a lot in common with other children		X	
I feel in tune with other children		X	
I feel like other children want to be with me		X	
I feel like that I usually fit in with other children around me		X	
When I want to do something for fun, I can usually find friends to join me		X	
When I am with other children, I feel like I belong		X	
*Family group integration*			
In my family, I feel part of a group of people that does things together		X	
I have a lot in common with people in my family			X
I feel in tune with the people in my family		X	
I feel like people in my family want to be with me		X	
I feel that I usually fit in with my family		X	
When I want to do something for fun, I can usually find people in my family to join me		X	
When I am with my family, I feel like I belong		X	

**Table 8:** Coding of Items of the Peer Network and Dyadic Loneliness Scale (PNDLS).

Item	Emotional Loneliness	Social Loneliness	No Loneliness
*Peer dyadic loneliness*			
Some kids have a friend that is always there for them when they need it BUT Other kids don’t have a friend that is always there for them when they need it	X		
Some kids have someone their age who is a really close friend BUT Other kids don’t have anybody their age who is a really close friend	X		
Some kids wish they had a friend that really cared about how they feel inside BUT Other kids feel like they already do have a friend that really cared about how they feel inside	X		
Some kids don’t have a friend that they can talk to about important things BUT Other kids do have a friends that they can talk to about important things	X		
Some kids don’t have anyone special their age to share things with BUT Other kids do have anyone special their age to share things with	X		
Some kids have a friend that they know will always care about them BUT Other kids just wish they had a friend that would always care about them	X		
Some kids hardly ever feel lonely because they have a best friend BUT Other kids wish they had a best friend so they wouldn’t feel so lonely		X	
Some kids with that someone their age thought they were really special BUT Other kids feel like someone their age already thinks they’re really special			X
*Peer network loneliness*			
Some kids feel like they really fit in with other kids BUT Other kids don’t feel like they fit in very well with other kids		X	
Some kids almost always feel left out when they’re with others their age BUT Other kids almost never feel left out when they’re with others their age		X	
Some kids hardly ever feel accepted by others their age BUT Other kids feel accepted by others their age most of the time		X	
Some kids really feel like they’re part of a group BUT Other kids feel like they’re not really part of a group		X	
Some kids are often bored when they’re with other kids BUT Other kids are hardly ever bored when they’re with other kids			X
Some kids usually have other kids to do things with BUT Other kids hardly ever have kids to do things with		X	
Some kids feel like most kids like them BUT Other kids feel like hardly any kids like them		X	
Some kids feel lonely a lot because they wish other kids included them more in things BUT Other kids don’t feel lonely very much because they think other kids usually do include them in things		X	

## Data Availability

Analysis scripts and data used in the analyses are accessible at https://osf.io/5wn93/.

## Appendix A

**Table A1:** Separate Regression Analyses for the Moderators Predicting Reliability Scores of the Children’s Loneliness Scale (CLS).

Moderator	*k*	*B*		*SE B*	95% CI		*F*	*df*	*p*
General population	319						0.89	5, 313	0.49
Non-clinical	252	−1.97		0.03	−2.02,	−1.92			
Physical health problems	17	−2.16		0.11	−2.37,	−1.94			
Physical health problems + controls	5	−1.99		0.19	−2.37,	−1.61			
Special education needs	19	−1.95		0.10	−2.14,	−1.75			
Special education needs + controls	19	−1.95		0.09	−2.13,	−1.76			
Mental health problems	7	−2.16		0.16	−2.48,	−1.85			
Age	319						5.38	1, 317	0.02
Children	212	−1.94	_a_	0.03	−1.99,	−1.88			
Adolescence	107	−2.05	_b_	0.04	−2.13,	−1.97			
Gender	307	−0.00		0.01	−0.02,	0.01	0.09	1, 305	0.77
Country	319						2.24	2, 316	0.11
US	153	−1.98		0.03	−2.04,	−1.91			
Non-US, Western	79	−1.90		0.05	−1.99,	−1.81			
Non-Western	87	−2.04		0.04	−2.12,	−1.95			
Translated	318						0.32	1, 316	0.57
No	202	−1.97		0.03	−2.02,	−1.91			
Yes	116	−1.99		0.04	−2.07,	−1.92			
Adaptation	292						20.6	3, 288	<0.001
Original scale	199	−2.09	_a_	0.03	−2.14,	−2.04			
Brief version	44	−1.82	_b_	0.06	−1.93,	−1.71			
Another number of response categories	26	−1.67	_b_	0.07	−1.82,	−1.52			
Another number of items and response categories	23	−1.64	_b_	0.08	−1.80,	−1.49			

*Note.* Effects sizes are significantly different if they do not have the same subscript. For the categorical variables, the given regression coefficients represent the mean effect sizes for each category. *k* = number of effect sizes; *B* = regression coefficient; CI = confidence interval.

**Table A2:** Separate Regression Analyses for the Moderators Predicting Reliability Scores of the Rasch-Type Loneliness Scale (RTLS).

Moderator	*k*	*B*		*SE B*	95% CI		*F*	*df*	*p*
Subscale	94						1.25	2, 91	0.29
Total loneliness score	51	−1.88		0.1	−2.02,	−1.75			
Emotional loneliness	22	−1.77		0.10	−1.97,	−1.57			
Social loneliness	21	−1.87		0.10	−2.07,	−1.67			
General population	94						4.57	2, 91	0.01
Non-clinical	83	−1.80	_a_	0.06	−1.91,	−1.69			
Physical health problems	5	−2.18	_a,b_	0.20	−2.58,	−1.78			
Special education needs, mental health problems, controls	6	−2.29	_b_	0.19	−2.67,	−1.92			
Age	94						4.49	3, 90	0.01
Adolescence	6	−1.81	_a,b_	0.18	−2.17,	−1.45			
Young adulthood	16	−2.19	_b_	0.12	−2.42,	−1.96			
Middle adulthood	35	−1.91	_a,b_	0.09	−2.08,	−1.74			
Old age	37	−1.68	_a_	0.08	−1.84,	−1.52			
Gender	84	0.44		0.32	−0.20,	1.07	1.89	1, 82	0.17
Country	94						0.56	2, 91	0.57
US	8	−1.72		0.10	−2.11,	−1.39			
Non-US, Western	79	−1.86		0.06	−1.98,	−1.74			
Non-Western	7	−2.04		0.21	−2.44,	−1.63			
Translated	91						3.47	1, 89	0.07
No	43	−1.96		0.08	−2.12,	−1.81			
Yes	48	−1.76		0.07	−1.91,	−1.61			
Adaptation	89						1.84	3, 85	0.15
Original scale	37	−1.90		0.07	−2.05,	−1.76			
Brief version	26	−1.70		0.10	−1.89,	−1.51			
Another number of response categories	16	−1.92		0.11	−2.14,	−1.71			
Another number of items and response categories	10	−1.65		0.13	−1.92,	−1.38			

*Note.* Effects sizes are significantly different if they do not have the same subscript. For the categorical variables, the given regression coefficients represent the mean effect sizes for each category. *k* = number of effect sizes; *B* = regression coefficient; CI = confidence interval.

**Table A3:** Separate Regression Analyses for the Moderators Predicting Reliability Scores of the Social and Emotional Loneliness Scale for Adults (SELSA).

Moderator	*k*	*B*		*SE B*	95% CI		*F*	*df*	*p*
Subscale	103						2.13	2, 100	0.12
Social loneliness	42	−1.85		0.08	−2.00,	−1.70			
Family loneliness	29	−1.93		0.08	−2.10,	−1.77			
Romantic loneliness	32	−2.00		0.08	−2.16,	−1.84			
General population	108						0.96	1, 106	0.33
Non-clinical	101	−1.93		0.06	−2.05,	−1.80			
Clinical	7	−1.67		0.25	−2.17,	−1.17			
Age	108						0.45	3, 104	0.72
Adolescence	13	−1.80		0.16	−2.12,	−1.48			
Emerging adulthood	62	−1.91		0.08	−2.07,	−1.74			
Young adulthood	26	−1.93		0.13	−2.18,	−1.68			
Middle adulthood	7	−2.14		0.25	−2.64,	−1.65			
Gender	107	0.67		0.36	−0.05,	1.40	3.40	1, 105	0.07
Country	107						1.91	2, 104	0.15
US	31	−1.88		0.11	−2.09,	−1.66			
Non-US, Western	56	−2.00		0.08	−2.17,	−1.84			
Non-Western	20	−1.66		0.16	−1.97,	−1.35			
Translated	107						9.57	1, 105	0.003
No	65	−2.05		0.07	−2.19,	−1.90			
Yes	42	−1.68		0.09	−1.87,	−1.49			
Adaptation	104						7.12	3, 100	<0.001
Original scale	19	−2.33	_b_	0.13	−2.58,	−2.07			
Brief version	70	−1.83	_a_	0.07	−1.96,	−1.69			
Another number of response categories	5	−2.21	_b_	0.18	−2.55,	−1.86			
Another number of items and response categories	10	−1.53	_a_	0.16	−1.84,	−1.21			

*Note.* Effects sizes are significantly different if they do not have the same subscript. For the categorical variables, the given regression coefficients represent the mean effect sizes for each category. *k* = number of effect sizes; *B* = regression coefficient; CI = confidence interval.

**Table A4:** Separate Regression Analyses for the Moderators Predicting Reliability Scores of the Differential Loneliness Scale (DLS).

Moderator	*k*	*B*		*SE B*	95% CI		*F*	*df*	*p*
Subscale	26						17.16	3, 22	<0.001
Romantic loneliness	6	−2.20	_c_	0.14	−2.50,	−1.90			
Friends loneliness	8	−1.51	_b_	0.12	−1.77,	−1.26			
Family loneliness	7	−1.95	_c_	0.13	−2.21,	−1.68			
Community loneliness	5	−0.95	_a_	0.15	−1.26,	−0.63			
Age	29						0.04	1, 27	0.83
Adolescence	24	−1.77		0.12	−2.01,	−1.54			
Adulthood	5	−1.71		0.26	−2.25,	−1.17			
Gender	29	0.19		0.51	−0.86,	1.24	0.14	1, 27	0.71
Country	27						0.68	1, 25	0.42
US	14	−1.65		0.16	−1.97,	−1.33			
Non-US, Western	13	−1.83		0.16	−2.17,	−1.50			
Translated	29						1.09	1, 27	0.31
No	14	−1.65		0.15	−1.96,	−1.34			
Yes	15	−1.87		0.14	−2.16,	−1.57			

*Note.* Effects sizes are significantly different if they do not have the same subscript. For the categorical variables, the given regression coefficients represent the mean effect sizes for each category. *k* = number of effect sizes; *B* = regression coefficient; CI = confidence interval.

**Table A5:** Separate Regression Analyses for the Moderators Predicting Reliability Scores of the Relational Provisions Loneliness Questionnaire (RPLQ).

Moderator	*k*	*B*	*SE B*	95% CI		*F*	*df*	*p*
Gender	12	15.69	5.40	3.65,	27.72	8.43	1, 10	0.02

*Note. k* = number of effect sizes; *B* = regression coefficient; CI = confidence interval.

**Table A6:** Separate Regression Analyses for the Moderators Predicting Reliability Scores of the Peer Network and Dyadic Loneliness Scale (PNDLS).

Moderator	*k*	*B*		*SE B*	95% CI		*F*	*df*	*p*
Subscale	16						1.95	1, 14	0.18
Peer network loneliness	8	−1.89		0.07	−2.05,	−1.74			
Peer dyadic loneliness	8	−1.79		0.07	−1.95,	−1.64			
Age	16						10.41	1, 14	0.01
Children	6	−1.69	_a_	0.06	−1.82,	−1.57			
Adolescence	10	−1.95	_a_	0.05	−2.06,	−1.84			
Gender	16	−0.30		0.32	−0.99,	0.38	0.90	1, 14	0.36
Country	16						0.94	1, 14	0.35
US	6	−1.77		0.10	−1.99,	−1.55			
Non-US	10	−1.90		0.09	−2.09,	−1.71			
Translated	16						0.94	1, 14	0.35
No	6	−1.77		0.10	−1.99,	−1.55			
Yes	10	−1.90		0.09	−2.09,	−1.71			
Adaptation	16						2.34	1, 14	0.15
Original scale	11	−1.91		0.07	−2.07,	−1.76			
Brief version	5	−1.73		0.09	−1.93,	−1.53			

*Note.* Effects sizes are significantly different if they do not have the same subscript. For the categorical variables, the given regression coefficients represent the mean effect sizes for each category. *k* = number of effect sizes; *B* = regression coefficient; CI = confidence interval.
